# Soluble epoxide hydrolase inhibitor enhances synaptic neurotransmission and plasticity in mouse prefrontal cortex

**DOI:** 10.1186/s12929-015-0202-7

**Published:** 2015-10-22

**Authors:** Han-Fang Wu, Hsin-Ju Yen, Chi-Chen Huang, Yi-Chao Lee, Su-Zhen Wu, Tzong-Shyuan Lee, Hui-Ching Lin

**Affiliations:** Institute and Department of Physiology, School of Medicine, National Yang-Ming University, Taipei, 11221 Taiwan; Ph.D. Program for Neural Regenerative Medicine, College of Medical Science and Technology, Taipei Medical University, Taipei, 11031 Taiwan; Center for Neurotrauma and Neuroregeneration, Taipei Medical University, Taipei, 11031 Taiwan; Department of Anesthesiology, Chi-Mei Medical Center, Tainan, Taiwan; Brain Research Center, National Yang-Ming University, Taipei, 11221 Taiwan

**Keywords:** Soluble epoxide hydrolase, Prefrontal cortex, Excitatory synaptic neurotransmission

## Abstract

**Background:**

The soluble epoxide hydrolase (sEH) is an important enzyme chiefly involved in the metabolism of fatty acid signaling molecules termed epoxyeicosatrienoic acids (EETs). sEH inhibition (sEHI) has proven to be protective against experimental cerebral ischemia, and it is emerging as a therapeutic target for prevention and treatment of ischemic stroke. However, the role of sEH on synaptic function in the central nervous system is still largely unknown. This study aimed to test whether sEH C-terminal epoxide hydrolase inhibitor, 12-(3-adamantan-1-yl-ureido) dodecanoic acid (AUDA) affects basal synaptic transmission and synaptic plasticity in the prefrontal cortex area (PFC). Whole cell and extracellular recording examined the miniature excitatory postsynaptic currents (mEPSCs) and field excitatory postsynaptic potentials (fEPSPs); Western Blotting determined the protein levels of glutamate receptors and ERK phosphorylation in acute medial PFC slices.

**Results:**

Application of the sEH C-terminal epoxide hydrolase inhibitor, AUDA significantly increased the amplitude of mEPSCs and fEPSPs in prefrontal cortex neurons, while additionally enhancing long term potentiation (LTP). Western Blotting demonstrated that AUDA treatment increased the expression of the N-methyl-D-aspartate receptor (NMDA) subunits NR1, NR2A, NR2B; the α-Amino-3-hydroxy-5-methyl-4-isoxazolepropionic acid (AMPA) receptor subunits GluR1, GluR2, and ERK phosphorylation.

**Conclusions:**

Inhibition of sEH induced an enhancement of PFC neuronal synaptic neurotransmission. This enhancement of synaptic neurotransmission is associated with an enhanced postsynaptic glutamatergic receptor and postsynaptic glutamatergic receptor mediated synaptic LTP. LTP is enhanced via ERK phosphorylation resulting from the delivery of glutamate receptors into the PFC by post-synapse by treatment with AUDA. These findings provide a possible link between synaptic function and memory processes.

## Background

A ubiquitous bifunctional vertebrate enzyme, soluble epoxide hydrolase (sEH), is comprised of a C-terminal epoxide hydrolase (EH) domain and an N-terminal lipid phosphatase (PT) domain, and is widely distributed in a variety of mammalian organs and tissues such as liver, vascular endothelium, kidney [1–5]. Soluble epoxide hydrolase (sEH) is the major key regulator involved in the epoxyeicosatrienoic acids (EETs) metabolism effects [6]. Inhibition of sEH metabolism through pharmacological inhibition, or genetic deletion, has been shown to effectively increase endogenous EET levels. Due to this activity, sEH inhibitors are being developed as antihypertensive agents to prevent cardiac hypertrophy, inflammation and hyperglycemia in diabetic mice [7–10]. Thus sEH inhibition (sEHI) can have a somewhat similar effect to EET augmentation.

In the brain, sEH distributed in the cortex, striatum, hypothalamus and brain stem regions [11, 12]. The sEH is expressed in neural tissue and cerebral arterioles [2, 11, 13]. Immunohistochemistry technique observed that sEH is co-localized with neuron in cerebral cortex and striatum in the stroke brain [4]. Several animal studies, sEH activity and sEH-EET pathway has been implicated in the disease associated neurotransmission imbalance such as anxiety-related behavior and seizure disorder [14–16]. Electrophysiology studies demonstrated that the C-terminal epoxide hydrolase (EH) inhibitor 12-(3-adamantan-1-yl-ureido) dodecanoic acid (AUDA) is similarly elevated the endogenous EETs to increase neuronal activity in the hypothalamus and brain stem of SHR rats [12]. The 5,6-EET increases the spontaneous excitatory postsynaptic current (sEPSC) frequency in spinal cord slices to affect the pain sensitivity [17]. Together, these studies raise the possibility that the EETs could modulate the physiological actions through the enhancement of excitatory neurotransmitter and neuronal activity. However, how the sEH-EET affect the basal levels of glutamate within prefrontal cortex remain unclear.

Glutamate, which is recognized as the major excitatory neurotransmission in the CNS [18], is involved in basal neurotransmission, synaptic plasticity, learning, and memory [19–21]. Activity dependent long term potentiation increases synaptic strength and requires a contribution from postsynaptic receptors such as α-Amino-3-hydroxy-5-methyl-4-isoxazolepropionic acid (AMPA) receptors and N-methyl-D-aspartate receptor (NMDA) receptors [22, 23]. NMDA receptor activation induces Ca^2+^ influx and triggers Ca^2+^/calmodulin-dependent protein kinase II (CaMKII) thereby enhancing synaptic strength [24, 25]. Mitogen-activated protein kinase (MAPK) and phophatidylinositol-3kinase (PI3K) are both implicated in long term potentiation (LTP) and cognitive learning [26–28]. The AMPA receptor trafficking delivery mechanism to synaptic membranes plays an important role in LTP [29, 30]. However, the effect and mechanisms by which sEH inhibitor, AUDA modulates synaptic plasticity remain unclear.

In current study, we aimed to test whether AUDA affects basal synaptic transmission and synaptic plasticity. Firstly, we determined the effects of AUDA on glutamatergic neurotransmission and synaptic efficacy in prefrontal cortex. Secondly, we further determined the expression of glutamate receptors and mechanisms underlying the AUDA-induced alteration in neurophysiology function. Finally, we wished to explore the possible signal pathway underlying AUDA-mediated enhancement of PFC glutamatergic neurotransmission. Here we found that the AUDA induces the enhancement of synaptic transmission, LTP and glutamate receptors in the prefrontal cortex. The sEH inhibition may increase the levels of EETs with subsequent activation of extracellular-signal-regulated kinases (ERKs) and modulation synaptic plasticity in the PFC area.

## Methods

### Animals

All procedures have been approved by the Institutional Animal Care and Use Committee of the College of Medicine, National Yang-Ming University (Taipei, Taiwan). Eight weeks old C57BL/6 mice were used in this study and housed four to a cage in a temperature-controlled (24 °C) animal colony under a 12:12 light/dark cycle, with lights on at 7:00 AM. Pelleted chow and water were available *ad libitum*. All experimental procedures took place during the light cycle.

### Brain slice preparation and electrophysiological recordings of the prefrontal cortex

Brain slices (400 μm thickness) were prepared as described previously [31]. Whole-cell recordings were made from the soma of visually-identified pyramidal-like neurons located in the PFC. Neurons were identified as projection neurons based on their intrinsic electrophysiological properties [32] in potassium gluconate-containing electrodes. The miniature excitatory postsynaptic currents (mEPSCs) were recorded in the presence of bicuculline (10 μM; Tocris) Tetrodotoxin TTX (1 μM; Tocris). We also examined paired pulse facilitation (PPF) in slices. The ratio of the amplitude of the first EPSP (fEPSP1) divided by the amplitude of the second EPSP (fEPSP2) was examined at 30 ms, 60 ms, 90 ms and 120-ms inter-pulse intervals. NMDA receptor dependent long term potentiation was induced by high frequency stimulation (HFS) protocols, 3 times of 1 s, 100Hz stimulus trains separated by a 1 min interval between trains [33].

### Drugs

AUDA, (±) 14 (15)- epoxy-5Z, 8Z, 11Z-eicosatrienoic acid (14,15-EET) and 14,15-epoxyeicosa-5 (Z)-enoic acid (14,15-EEZE) were obtained from Cayman. The stock solution was prepared in dimethyl sulphoxide (DMSO). The perused concentration of DMSO did not exceed 0.1 % and had no effect on basal synaptic transmission. In the present study, the vehicle control was 0.1 % DMSO in ACSF.

### Western blotting assay

Brain tissues were dissected and lysed in a lysis buffer containing 1 % Triton X-100, 0.1 % SDS, 50 mM Tris-HCl, pH 7.5, 0.3 M sucrose, 5 mM EDTA, 2 mM sodium pyrophosphate, 1 mM sodium orthovanadate, 1 mM enylmethylsulfonyl fluoride, supplemented with a complete protease inhibitor cocktail. Following sonication, lysates were centrifuged at 12,000 rpm for 30 min to obtain supernatants. The protein concentration of supernatants was measured using a Bradford assay and equal amount of protein were separated by SDS-PAGE electrophoresis, transferred to Immobilon-P membranes (Millipore). and incubated in 5 % nonfat dry milk for 60 min. Western blot analysis used GluR1 (1: 1000; Abcam, Cambridge, UK), GluR2^−^ (1: 1000; Abcam, Cambridge, UK) and β- Actin (1:1000; Millipore, Billerica, MA, USA), NR1 (1: 1000; Abcam, Cambridge, UK), NR2A (1: 1000; Millipore, Billerica, MA, USA) ,NR2B (1: 2000; Millipore, Billerica, MA, USA), D2 receptor (1: 1000; Millipore, Billerica, MA, USA,), ERK (1: 2000; Cell signaling  Technology, Boston, MA, USA), p-GluR1 (1: 1000; Abcam, Cambridge, UK), p-NR2B (1: 1000; Cell signaling Technology, Boston, MA, USA) antibody, reacted overnight at 4 °C, and then incubated with HRP-conjugated secondary antibodies for 1 h at room temperature. Immunoreactivity was detected by ECL Plus detection reagent (PerkinElmer, Boston, MA, USA). Films were exposed at different times to ensure the optimum density but not saturated and followed by densitometry. Protein levels were first normalized to internal control levels for each sample and then were measured as fold changes with respect to controls.

### Reverse transcription (RT) and quantitative (q) real-time polymerase chain reaction (PCR)

Total RNA was isolated using the TRIzol RNA extraction kit (Invitrogen), and 0.5 μg of RNA was subject to reverse transcription-PCR with M-MLV (Invitrogen). The specific PCR oligonucleotides for these NMDA and AMPA subunits, and β-actin as internal control listed as followed:

- NR1-F:5’-CTCATCTCTAGCCAGGTCTA-3’
- NR1-R: 5’-TCGCATCATCTCAAACCAGAC-3’
- NR2A-F: 5’-ACTCCACACTGCCCATGAAC-3’
- NR2A-R: 5’-TTGTTCCCCAAGAGTTTGCTT-3’
- NR2B-F: 5’-GTTTGATGAAATCGAGCTGGC-3’
- NR2B-R: 5’-TCCAGTTCCTGCAGGGAGTT-3’
- GluR1-F: 5’-AGGTTTGCTTTGTCACAA-3’
- GluR1-R: 5’-CTTCTCCAGGTC CTGAAA -3’
- GluR2-F: 5’-ATCAAGAAGCCTCAGAAGTCCAAA -3’
- GluR2-R: 5’-CTGACCCCAATGTAGGCAAAC-3’
- β-actin-F: 5’-TACAACCTCCTTGCAGCTCC-3’
- β-actin-R: 5’-ACAATGCCGTGTTCAATGG-3’

The PCR products were separated by 1 % agarose-gel electrophoresis and visualized with ethidium bromide staining. Quantitative RT-PCR was carried out using the StepOnePlus™ Real-Time PCR Systems and KAPA SYBR FAST qPCR Master Mix according to the manufacturer's instructions. In brief, β-actin was used as the internal control for quantitation of the expression of target genes in samples from vehicle control vs. AUDA-treated brain tissue. 

### Statistical analysis

All values are expressed as the mean ± SEM. Electrophysiology responses and the protein level of vehicle control and AUDA-exposed group, were assessed using the Student’s *t*-test. The stimulus-response (input–output) relationships, PPF and LTP analyzed by One-way ANOVA or Two-way ANOVA was followed with Bonferroni’s *post hoc* test. The difference between treated groups was considered significant if *p* < 0.05.

## Results

### Effects of sEH inhibitor AUDA on prefrontal cortex neurons

**Fig. 1:**
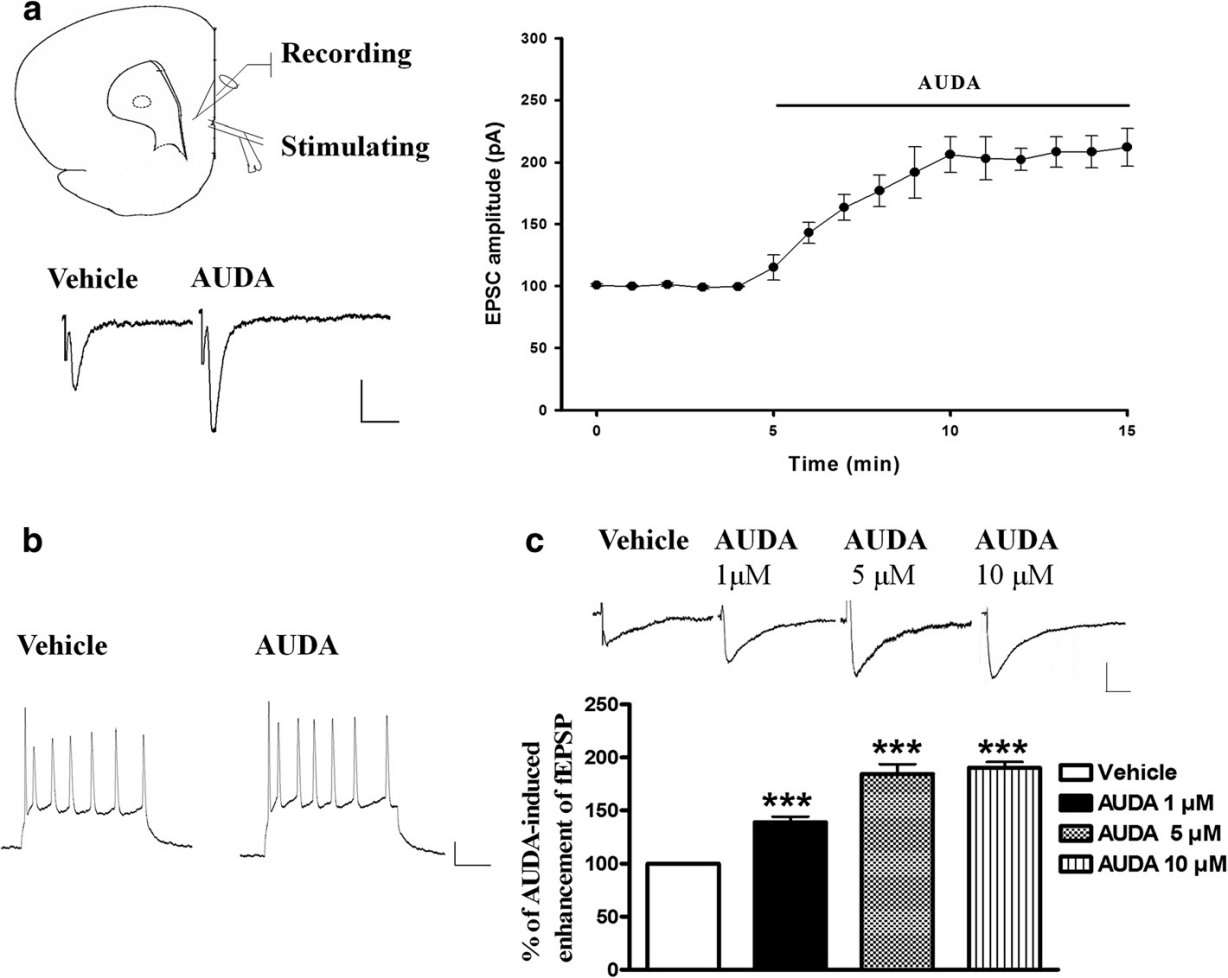
The sEH inhibitor, AUDA, alters synaptic response in PFC pyramidal neurons. **a** Schematic illustration of prefrontal cortex slices and placement of electrodes. The stimulating electrode was placed on layer II and the recording electrode was placed on layer V. Evoked EPSC was recorded of AMPA EPSC which was isolated in the presence of NMDA antagonist (APV 50 μM) and GABA_A_ receptor (bicuculline 10 μM) antagonists. (scale 40 ms, 50 pA). Time course showed that treatment of 10 μM AUDA increased the EPSC amplitude. **b** Traces showed that the number of spikes evoked by a current pulse during control/ACSF and perfusion with 10 μM AUDA (scale 100 ms ,20 pA). **c** The percentage of fEPSP response with 1 μM, 5 μM or 10 μM AUDA treatment. ***p < 0.001 vs. vehicle control

We assessed effect of sEHI on both basic neuronal features and excitatory synaptic transmission in the PFC. We utilized the protocol that othodromic stimuli applied to the layer II of rat prefrontal cortex slices elicited excitatory postsynaptic currents (EPSCs) in layer V. Superfusion of AUDA for 10 min resulted in significantly increased EPSC amplitudes (Fig. 1a). This result indicated that sEHI enhanced excitatory synaptic transmission in the PFC. To examine whether the intrinsic excitability of PFC neurons is changed by treatment of the sEH inhibitior AUDA, whole-cell patch clamp recordings were performed on PFC slices. The pyramidal neurons in layer V neurons have an average resting membrane potential of -61 ± 1.6 mV and an input resistance of 107 ± 7.8MΩ in the vehicle control, and an average resting membrane potential -60 ± 2.3 mV with an input resistance of 111 ± 5.2MΩ after AUDA treatment (*n* = 5). As shown in Fig. 1b, there is no difference between the vehicle control state and AUDA treatment in the number of action potentials that could be elicited in response to 150-pA depolarizing current pulses. Thus, AUDA treatment cannot significantly alter either the resting membrane potential or the input resistance. Application of different concentrations of AUDA, i.e. 1, 5,10 μM enhanced the initial response of fEPSPs by 138.9 ± 5.8 % (*n* = 5),184.8 ± 10.2 % (*n* = 5) and 190 ± 5.8 % (*n* = 5) of baseline respectively (Fig. 1c). Thus perfusion with 1, 5,10 μM of AUDA significantly enhanced the fEPSPs compared to vehicle control (F(3,16) = 36.3 P < 0.001).

### Effects of epoxyeicosatrienoic acids (EETs) on prefrontal cortex neurons

**Fig. 2:**
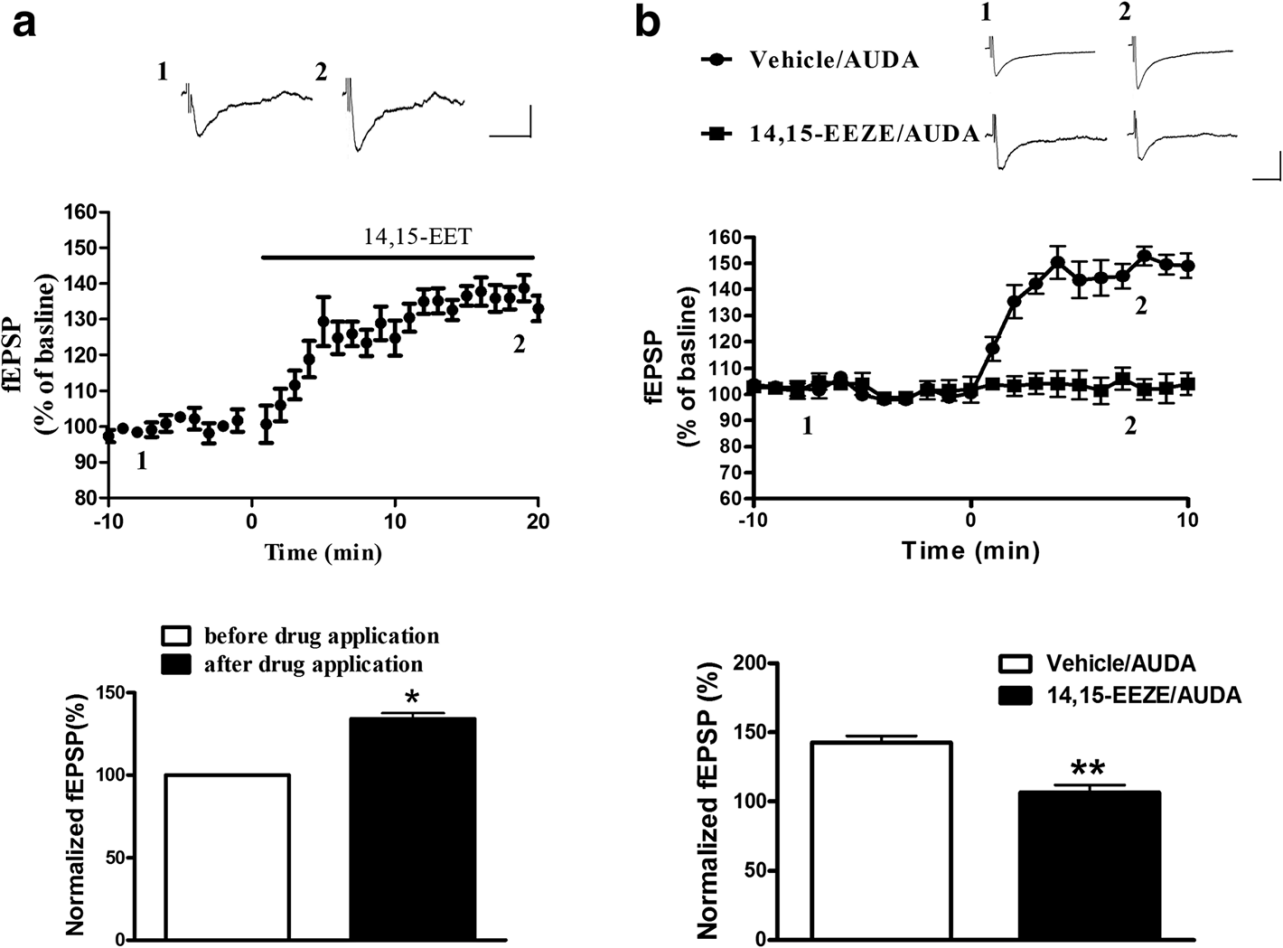
Enhancement of synaptic response by EETs at prefrontal cortex synapse. **a** PFC slice was perfused with 14,15-EET (10 μM, *n* = 5), and the percentage of fEPSPs response was measured. **b** The percentage of fEPSPs response was measured in the presence of vehicle combination with AUDA or 14,15-EEZE in combination with AUDA. *p < 0.05 vs. control, **p < 0.01 vs. vehicle/AUDA group

The enhancement of fEPSPs by sEH inhibitor could be due to its presence and to further promote the levels of EETs. Thus we determined whether the 14,15-EET produce similar postsynaptic glutamatergic neurotransmission effect of AUDA. We found that fEPSPs was significantly increased during 14,15-EET-treated (134.2 ± 12.1 %, *n* = 5 ) of baseline at PFC synapse (Fig. 2a). We further examined whether selective EET antagonist could inhibit the enhancement of fEPSPs by AUDA. The selective EET antagonist 14, 15-EEZE (1 μM) (*n* = 5) combination with AUDA (5 μM) (*n* = 5) were added into the PFC slice. As Shown in Fig. 2b, enhancement of fEPSPs by AUDA was dramatically inhibited by combining with 14, 15-EEZE at 1 μM 104.2 ± 3.1 %, *n* = 5). Thus AUDA induces enhancement of fEPSPs via increasing levels of EETs.

### AUDA enhances the I-O efficiency and glutamatergic transmission at PFC synapses

**Fig. 3:**
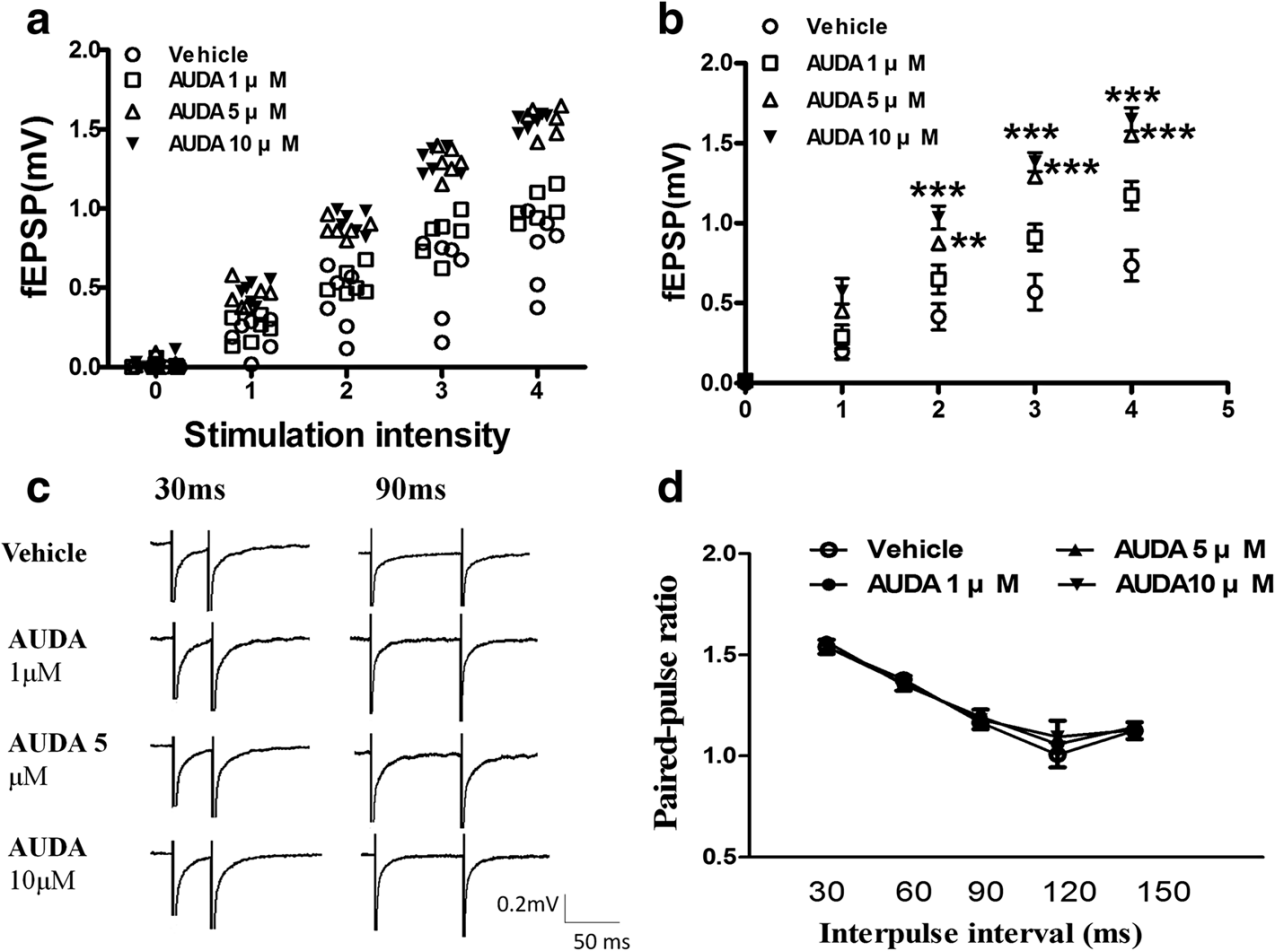
The sEH inhibitor, AUDA, increases postsynaptic efficiency of I-O glutamatergic transmission but not presynaptic glutamate release in PFC slice. **a** The I-O relationship was measured by the stimulation intensity and amplitude of fEPSP in the different concentration of 1 μM, 5 μM or 10 μM AUDA. **b** Mean value of I-O curves of glutamatergic transmission in PFC slices. **c** Sample traces were an average of 5-10 successive responses. The paired-pulse fEPSPs were evoked with intervals of 30 ms and 90 ms. Calibration; 50 pA, 30 ms. **d** Paired-pulse ratio was evoked at 30 ms, 60 ms, 90 ms, 120 ms, 150 ms intervals in the different concentration of AUDA. **p < 0.01, ***p < 0.001 vs. vehicle control

To determine whether the enhancement of fEPSPs was due to the alteration of the glutamate neurotransmitter mediated basal synapse response, we tested the relationship between the strength of a stimulus and its effect on response (input–output, IO) for fEPSPs in PFC neurons. The data showed that higher doses of AUDA induced larger fEPSPs responses which are determined at 2× threshold than vehicle control state (Fig. 3a). Different stimulation intensities were applied to observe the amplitude of the fEPSPs (Fig. 3b; two-way ANOVA, effect of treatment *F*_(3,96)_ = 23.37, *p* < 0.001; effect of stimulus intensity *F*_(4,96)_ = 244.2, *p* < 0.001; interaction *F*_(12,96)_ = 14.07, *p* < 0.001), and these results showed that AUDA enhanced the basal synaptic neurotransmission.

To determine whether AUDA treatment alters the presynaptic efficiency of excitatory synaptic transmission, we tested the PPF in PFC slices. The ratio of the fEPSP amplitude of the second fEPSP to the amplitude of the first fEPSP was examined at 30, 60, 90, 120 and 150 interpulse intervals (Fig. 3c). There was no difference in presynaptic glutamate release probability with AUDA treatment in PFC neurons (Fig. 3d, two-way ANOVA, effect of treatment *F*_(3,76)_ = 0.19, *p* = 0.92; effect of stimulus intensity *F*_(4,76)_ = 34.2, *p* < 0.001; interaction *F*_(12,76)_ = 0.21, *p* =0.99). Hence the effect of AUDA in PFC did not result in the alteration of presynaptic probability of neurotransmitter release.

**Fig. 4:**
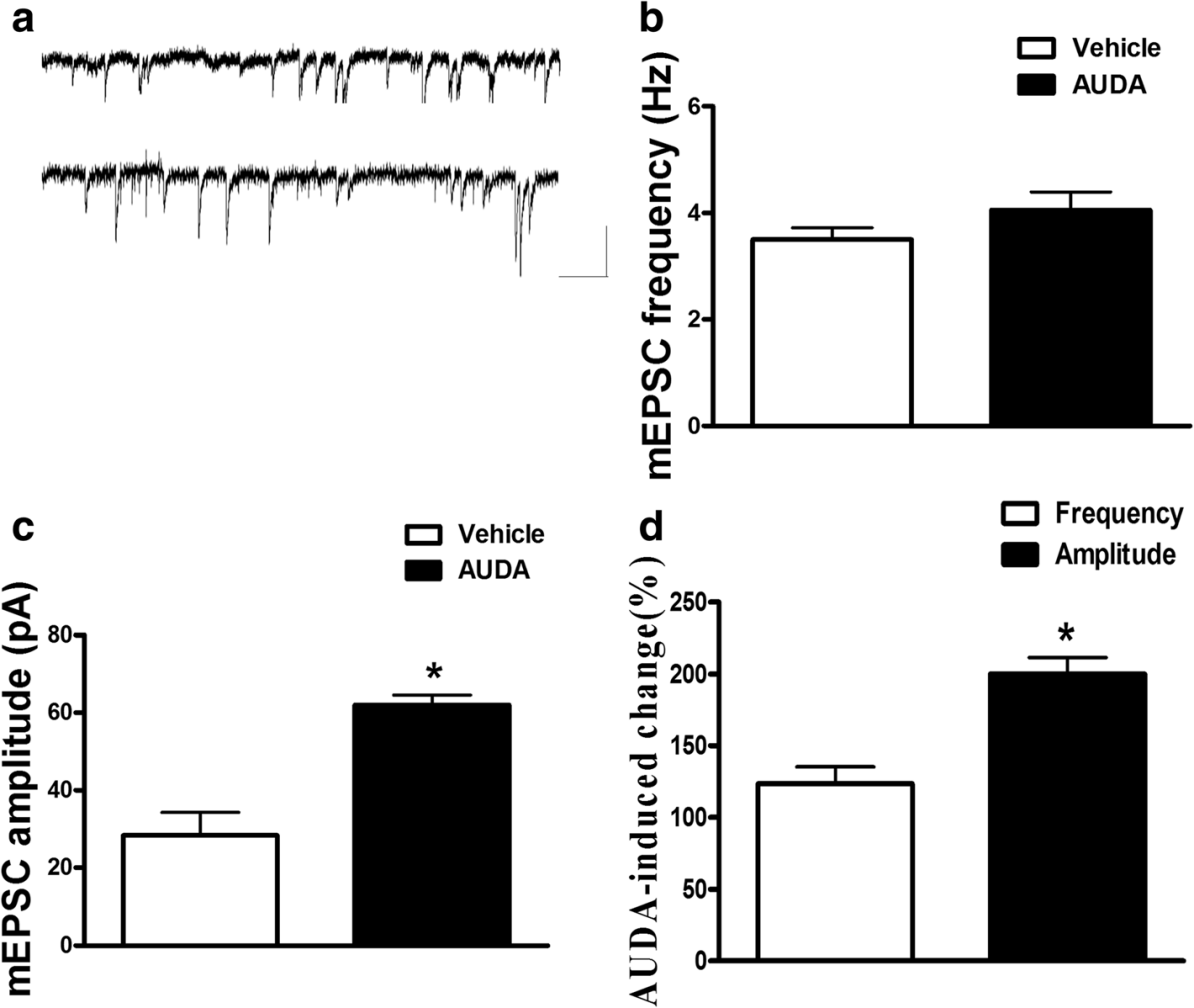
Enhancement of miniature excitatory postsynaptic current (mEPSC) amplitude in the PFC neurons by AUDA treatment. **a** Sample traces of mEPSCs were taken from brainslices of vehicle control, AUDA treatment in PFC neurons. mEPSCs were recorded in the PFC neurons at a holding potential of -70 mV in the presence of TTX (1 μM). Calibration: 50 pA, 100 ms. (**b** and **c**) The summary frequency and amplitude were measured for vehicle control and AUDA groups. (**d**) The AUDA-induced changes were measured in percentage of frequency-/amplitude- of mEPSCs response at 10 μM of AUDA *p < 0.05 vs. vehicle control

We further investigated the alteration of basal excitatory synaptic transmission in PFC neurons by AUDA treatment. PFC slices were made and whole-cell recordings were made from the soma of visually identified pyramidal-like neurons located in the PFC. Figure 4a, b shows that AUDA (10 μM) treatment resulted in a significantly higher mEPSCs amplitude compared to vehicle state (*p* < 0.05). The mean amplitude of the mEPSCs in the vehicle and AUDA treated groups was 28.4 ± 5.9 pA and 62.0 ± 2.6 pA (*p* < 0.05) respectively (Fig. 4c, d). The mean frequencies of mEPSCs in the vehicle and AUDA treated groups were 3.5 ± 0.3 Hz and 4,1 ± 0.4 Hz (Fig. 4b). These results demonstrated that enhancement of mEPSCs amplitude was observed in AUDA treated PFC slices, and the frequency of mEPSCs was not affected.

### AUDA enhances the long term potentiation (LTP) in the PFC neurons

**Fig. 5:**
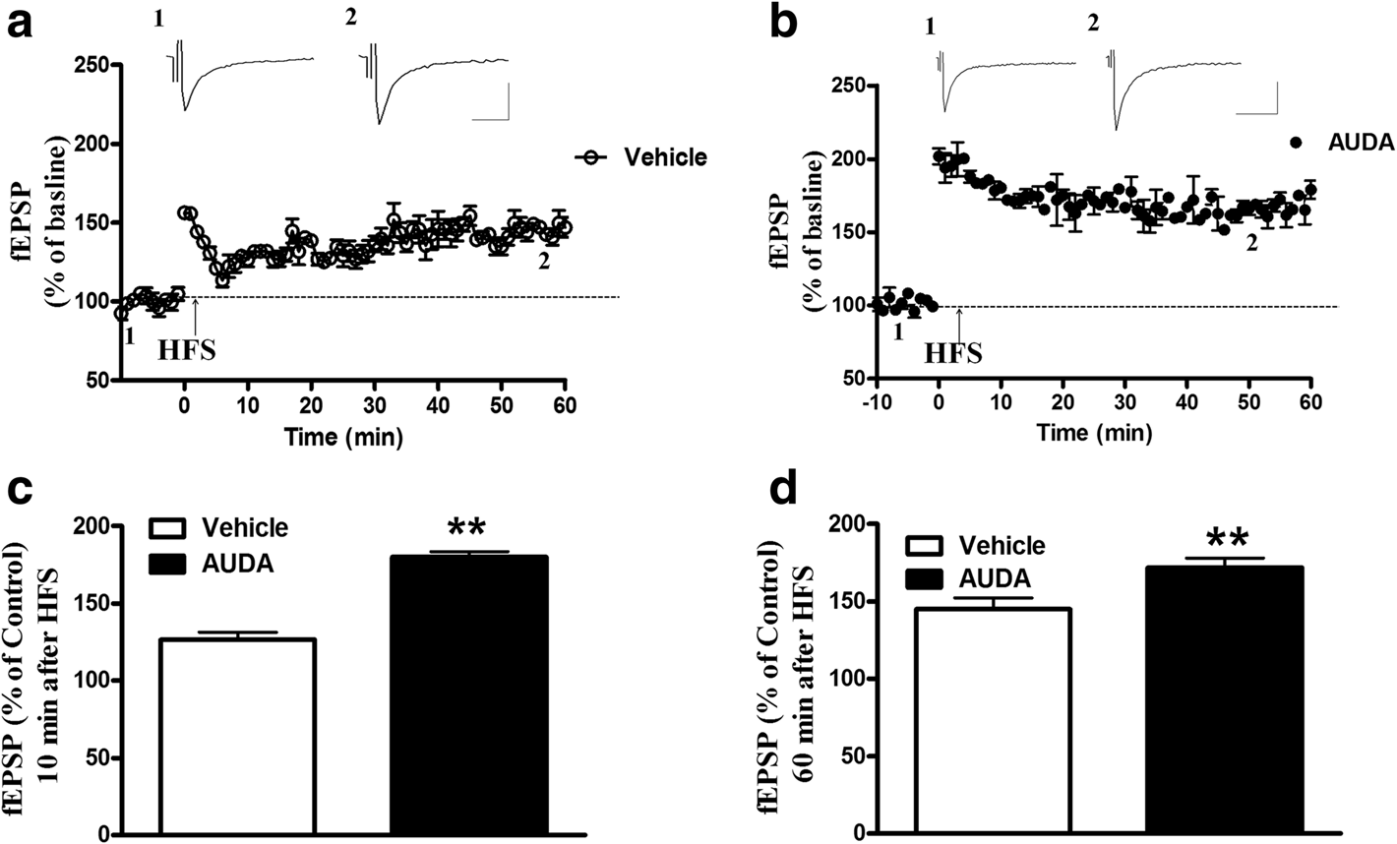
sEH inhibitor, AUDA, facilitates LTP in PFC slice. **a** The graph represents the mean ± SEM slope of fEPSPs plotted against time. Applied with 3× high-frequency stimulation of 100 Hz for 1 s induced LTP. **b** In the presence of 1 μM AUDA, the enhancement of LTP was observed. **c** Comparison of fEPSPs slope potentiation 10 min after tetanus in the absence or presence AUDA is shown. **d** Comparison of fEPSPs slope potentiation 60 min after tetanus in the absence or presence AUDA is shown. **p < 0.01 vs. vehicle control

We investigated if sEH inhibition was required for, or involved in, long term potentiation. We confirmed that applied high frequency stimulation (3 times for 1 sec at 100Hz stimuli separated by interval of 1 min; HFS) induced long term potentiation in PFC neurons. As shown in Fig. 5a, we used HFS protocol successfully induced LTP. The degree of HFS-induced LTP was enhanced in the presence of AUDA (Fig. 5b). We further compared the amplitude of synapse at 10 or 60 min following tetanus HFS treatment in the absence or presence of AUDA. The results showed a higher response for fEPSPs at 10 min after HFS (vehicle: 126.3 ± 4.8 % of baseline, *n* = 10 from 5 rats. AUDA: 180.3 ± 3.5 % of baseline, *n* = 10 from 6 rats.) which maintained LTP at 60 min after HFS (vehicle: 145.2 ± 7.4 % of baseline, *n* = 10 from 5 rats. AUDA: 172.1 ± 6.3 % of baseline, *n* = 10 from 6 rats) in the presence of AUDA (Fig. 5c, d). The results suggest that AUDA can modulate synaptic efficiency to facilitate the formation of and subsequently maintain LTP (*p* < 0.01).

### The expression of glutamate receptors is increased by the inhibition of sEH

**Fig. 6:**
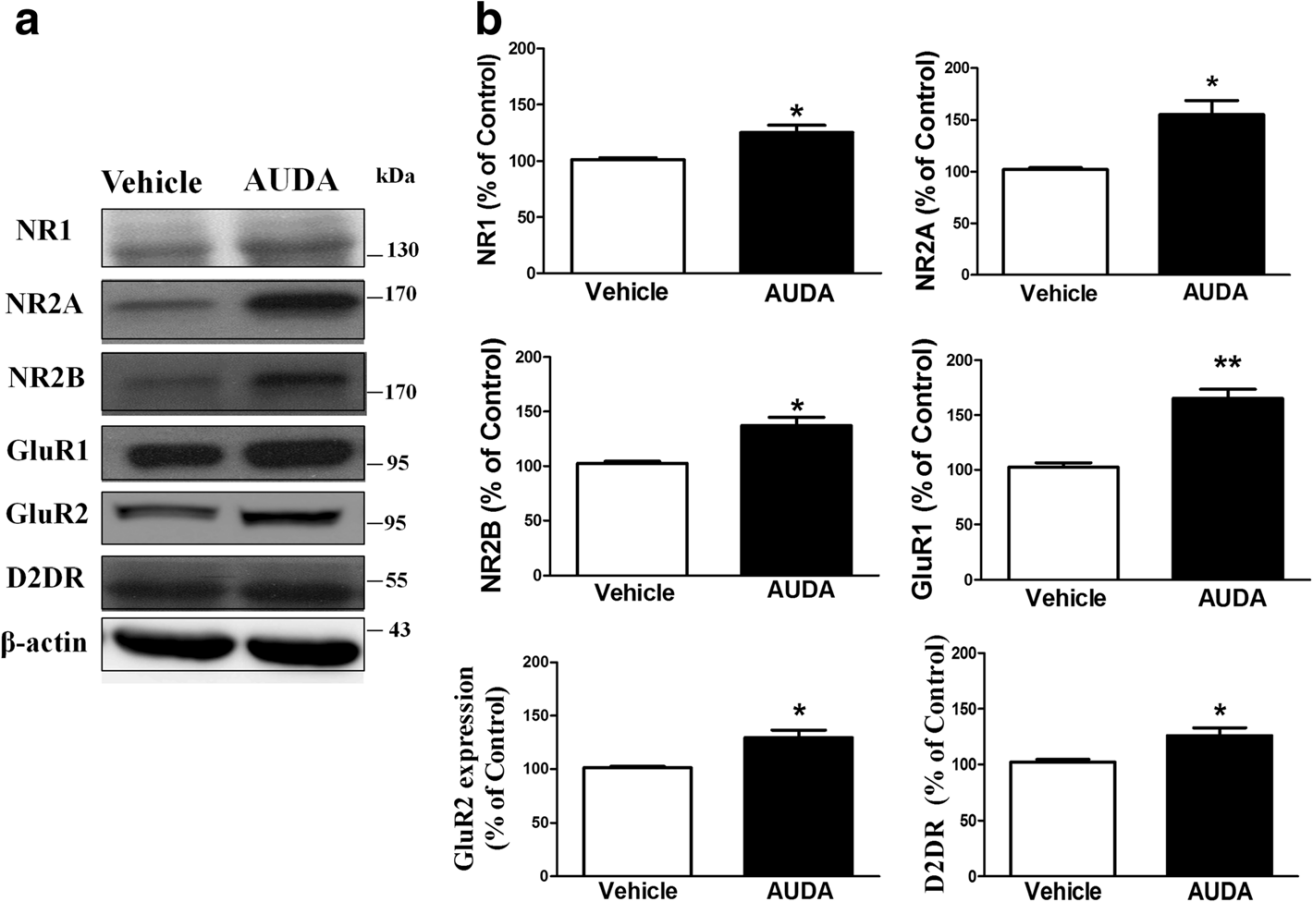
Synaptic receptors expression in PFC slices could be increased by the sEH inhibitor, AUDA. **a** The PFC slices were incubated with AUDA (10 μM) for 10 min and then washed to remove AUDA. One hour later, homogenate from the PFC was prepared and blotted with antibodies AMPA receptors subunit GluR1, GluR2, NMDA receptor subunits NR1, NR2A, NR2B and dopamine receptor D2 in the PFC slices. **b** The bar graph showed the normalized band intensity of synaptic receptors in PFC slice. *p < 0.05, **p < 0.01 vs. vehicle control

PFC glutamatergic neurotransmission is increased by inhibiting sEH. This may be caused by the alteration of glutamate receptor expression and function [34–37]. We determined whether the expression of AMPA receptors and NMDA receptors were altered by AUDA. Rats were decapitated and PFC slices were prepared. Slices were incubated with AUDA (10 μM) for 10 min and washed to remove the drug. The protein levels of the NMDA receptor were: NR1 (121.1 ± 6.6 % of vehicle, *n* = 5), NR2A (158.3 ± 13.7 % of vehicle, *n* = 5) and NR2B (135.2 ± 9.3 % of vehicle, *n* = 5). The levels of AMPA receptor GluR1 subunit (167.3 ± 10.4 % of control, *n* = 5) and GluR2 subunit (129.9 ± 10.9 % of vehicle, *n* = 5) were significantly higher in the AUDA-exposed than in the Vehicle control state (*p* < 0.05 or *p* < 0.01). The dopamine D2 receptor has been reported to be able to contribute to the enhanced synaptic efficacy of the hippocampus by co-treating with cocaine and high frequency stimulation [38]. We also found that AUDA treatment significantly increased the protein level of the dopamine D2 receptor (*n* = 5) (Fig. 6a, b) (*p* < 0.05).

**Fig. 7:**
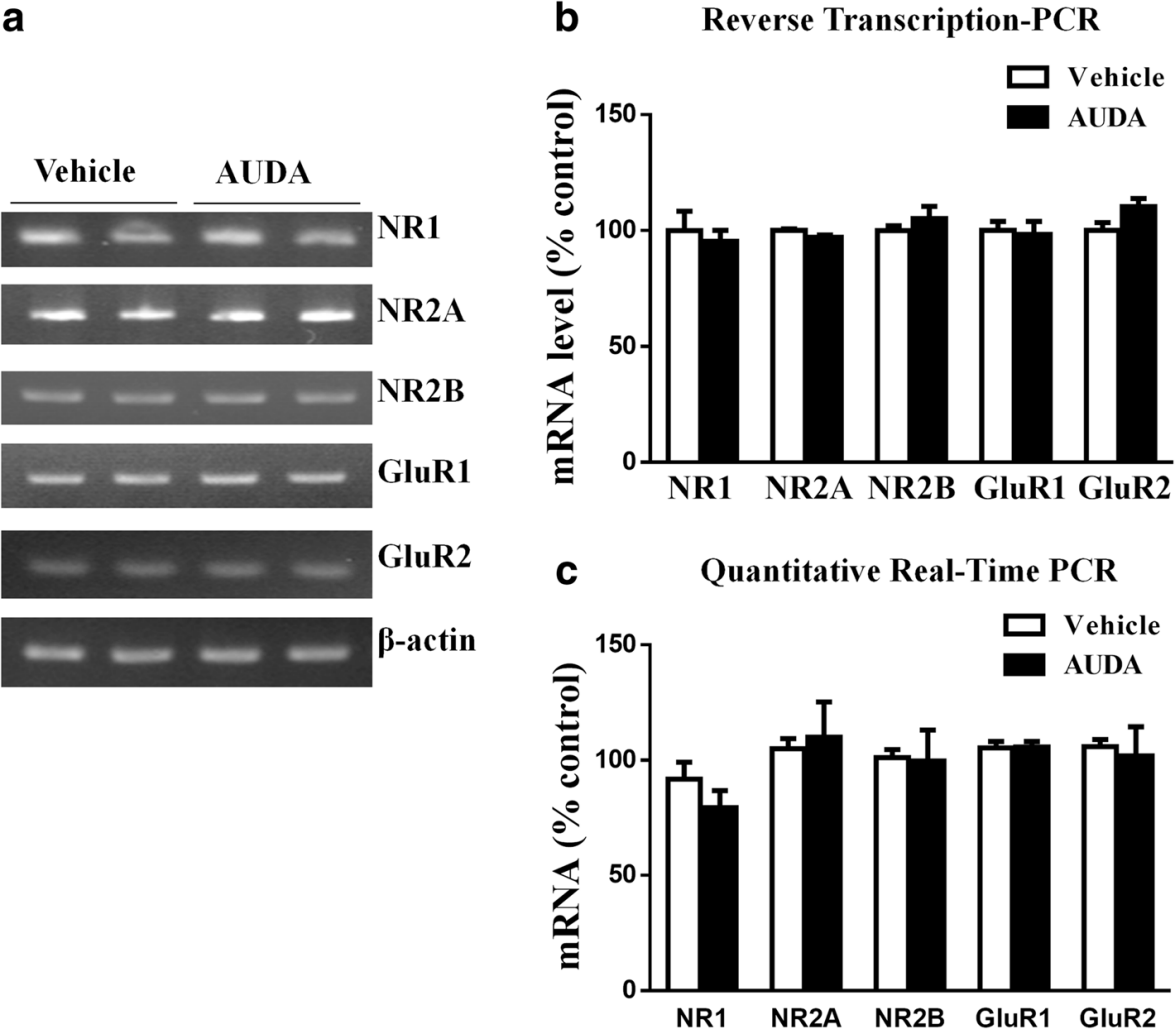
The sEH inhibitor AUDA does not affect the total amount of NR1, NR2A, NR2B, GluR1, GluR2 mRNA levels. **a**, **b** Total RNA was isolated from the PFC slices by treating with AUDA and mRNA expression for NR1, NR2A, NR2B, GluR1, GluR2 were analyzed with RT-PCR and (**c**) Real-Time qPCR. The mRNA expression for β-actin was used as an internal control

One possibility for the increase in NR1, NR2A, NR2B, GluR1, GluR2 levels is the increment in total protein expression. We therefore performed RT-PCR and real time qPCR to measure NR1, NR2A, NR2B, GluR1, GluR2 mRNA in tissue homogenates from PFC. By using RT-PCR, the mRNA levels of NR1, NR2A, NR2B, GluR1, GluR2 were: NR1 (95.3 ± 6.6 % of vehicle, *n* = 3), NR2A (97.1 ± 1.4 % of vehicle, *n* = 3) and NR2B (105.2 ± 7.4 % of vehicle, *n* = 3). The levels of GluR1 (98.3 ± 7.9 % of vehicle, *n* = 3) and GluR2 (110.3 ± 4.9 % of vehicle, *n* = 3) (Fig. 7a, b). Real-Time qPCR measured NR1 (96.6 ± 3.5 % of vehicle, *n* = 3), NR2A (112.8 ± 16.5 % of vehicle, *n* = 3) and NR2B (93.2 ± 12.9 % of vehicle, *n* = 3). The levels of GluR1 (104.6 ± 4.9 % of vehicle, *n* = 3) and GluR2 (98.3 ± 16.9 % of vehicle, *n* = 3) (Fig. 7c). The mRNA levels of NR1, NR2A, NR2B, GluR1, GluR2 examination showed no changes after AUDA treatment compared to vehicle control group.

**Fig. 8:**
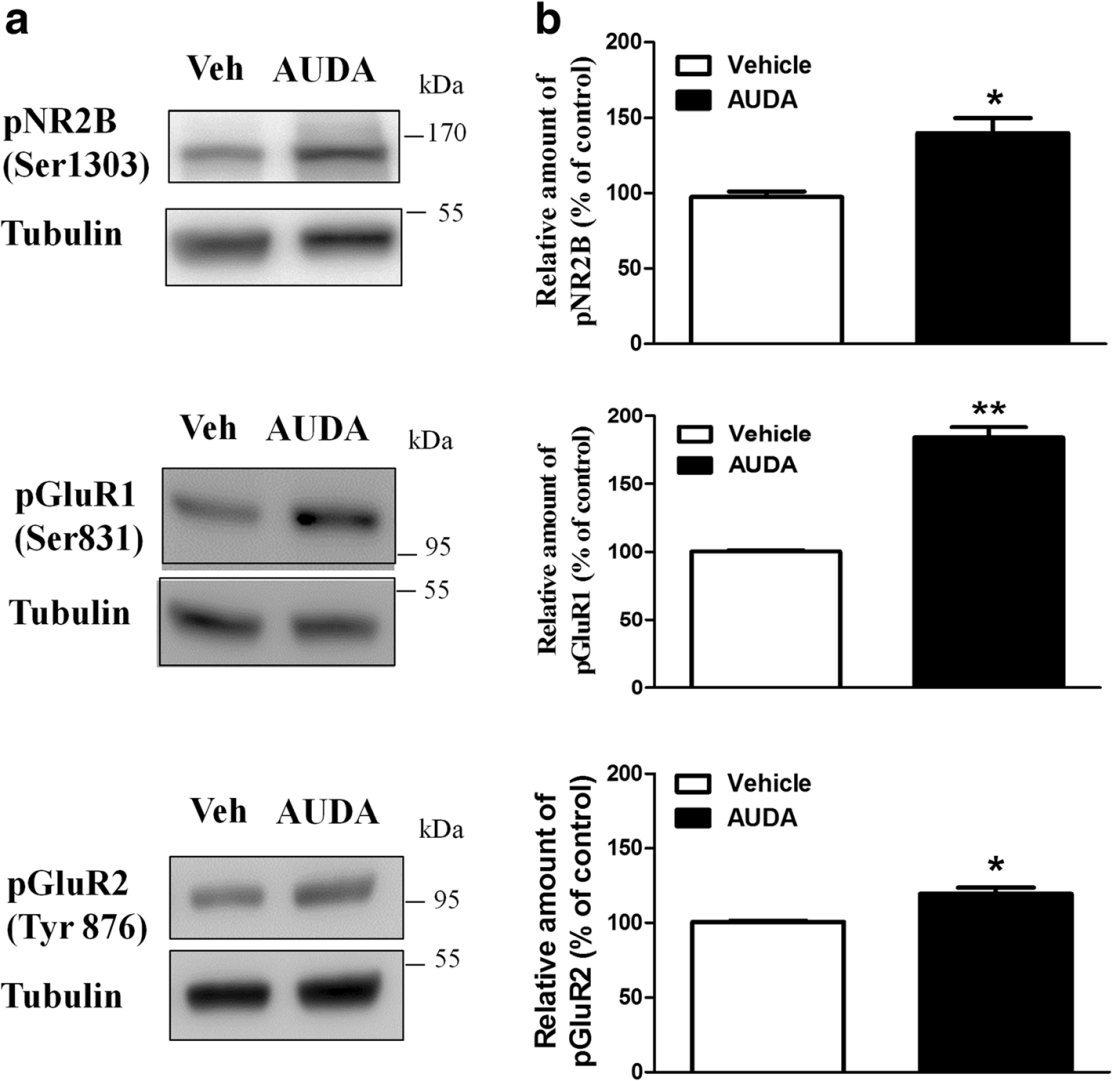
The sEH inhibitor AUDA increases the phosphorylation of protein levels by AUDA. **a** The tissue lysate of PFC area was prepared and blotted with antibodies against the phosphorylated  Ser1303 on NR2B, phosphorylated  Ser831 on GluR1 and phosphorylated  Tyr876 on GluR2. **b** The bar graph showed the normalized band intensity of phosphorylated synaptic receptors in PFC slice. *p <0.05, **p < 0.01 vs. vehicle control

We further confirmed that the increased protein levels are due to post-translational modification. We examined the phosphorylation of NR2B, GluR1, GluR2 in the PFC region treatment with AUDA. In the presence of AUDA increased NR2B phosphorylation (139.9 ± 9.9 % of vehicle, *n* = 4), GluR1phosphorylation (184.2 ± 7.6 % of vehicle, *n* = 4) and GluR2 phosphorylation (119.4 ± 4.1 % of vehicle, *n* = 4) (Fig.8).

**Fig. 9:**
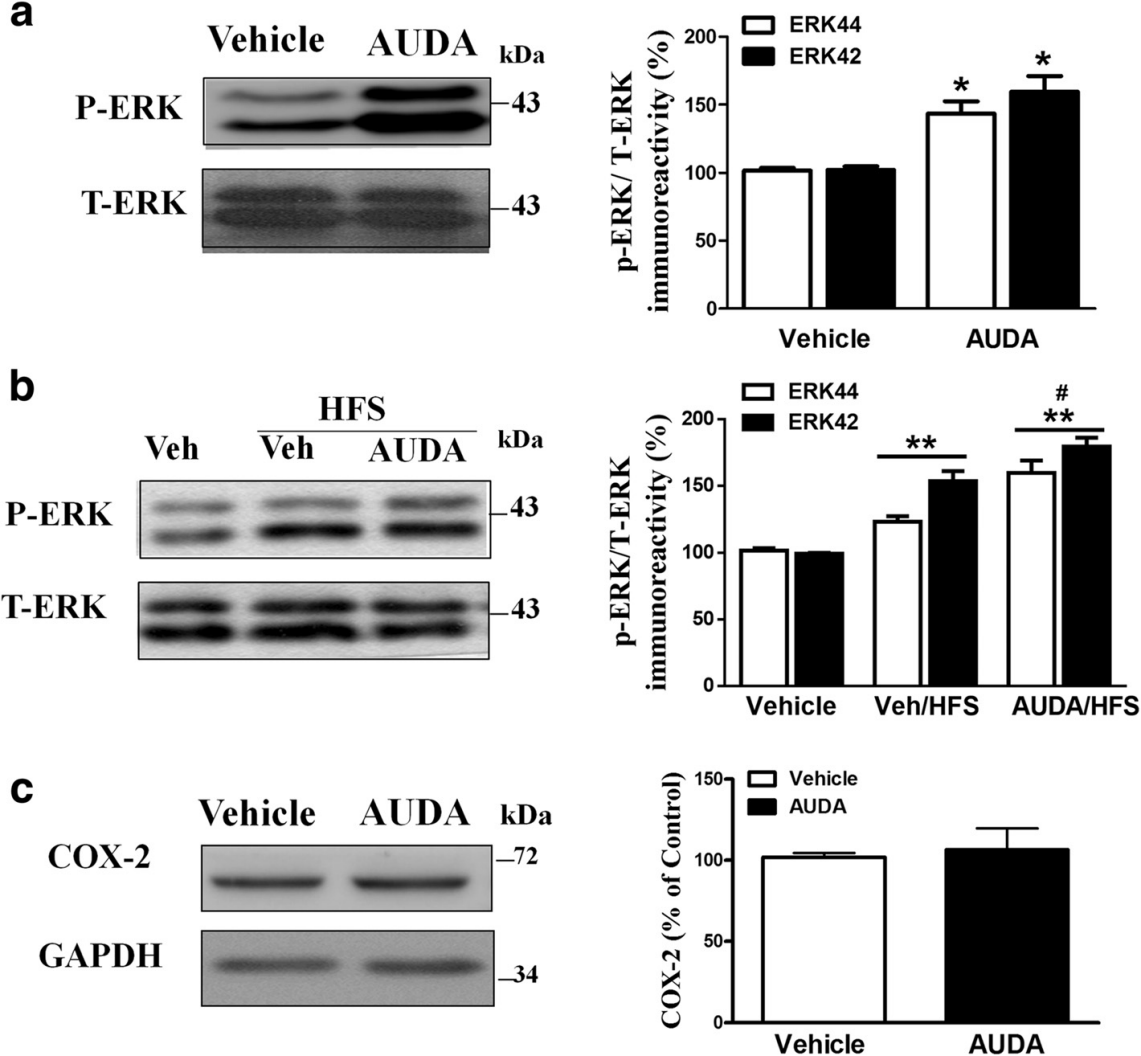
The sEH inhibitor AUDA induced the phosphorylation of ERK in PFC slices. **a** The PFC slices were incubated with AUDA (10 μM) for 10 min and then washed to remove AUDA. One hour later, homogenate from the PFC was prepared and blotted with antibodies directly against the active form of ERKs. AUDA-induced increase of ERKs phosphorylation in the PFC slices is shown. AUDA treatment increased the phosphorylated levels of ERK42 and ERK44 relative to the vehicle controls (ACSF). No change was observed when the cellular extract was blotted with an antibody that recognizes total ERKs, suggesting that the observed pERKs increments were not due to an increase in the total amount of ERKs. **b** Here shows the phosphorylated levels of ERK42 and ERK44 among the vehicle control, vehicle/HFS and AUDA/HFS groups. **c** The homogenate from the PFC following AUDA treatment was prepared and blotted with antibodies directly against the COX-2. GAPDH was the internal control *p < 0.05, **p < 0.01 vs. vehicle control; #*p* <0.05 vs. vehicle HFS group

We wished to explore the possible signal pathway underlying AUDA-mediated enhancement of PFC glutamatergic neurotransmission. Many studies have demonstrated that the EETs attenuated NF-κ B activation and increased PI3K/Akt and p42/p44 MAPK signaling in endothelial cells and cardiomyocytes [39]. Thus sEH inhibition may increase the levels of EETs with subsequent activation of ERK44/42 and modulation synaptic plasticity in the PFC area. As shown in 9A, the phosphorylated levels of ERK44 (140.8 ± 10.5 %, *n* = 5 ) and ERK42 (158.1 ± 14.7 %, *n* = 5) were significantly increased by AUDA treatment compared with vehicle control (*p* < 0.05) (Fig. 9a). The total ERKs did not change as a result of AUDA-treatment compared with vehicle control. These data indicated that AUDA could induce ERK44/42 activation in PFC region.

Our results demonstrated that the degree of HFS-induced LTP was enhanced in the presence of AUDA (Fig. 5b). We therefore tested whether AUDA combination with HFS could further induce ERK44/42 activation in PFC region. The phosphorylated degree of ERK44/42 (ERK44: 123.1 ± 4.7 % *n* = 5; ERK42: 153.6 ± 8.4 %, *n* = 5 ) was increased with LTP. The phosphorylated levels of ERK44/42 were further increased in the presence of AUDA combination with HFS-induced LTP protocol (ERK44: 159.8 ± 10.3 % *n* = 5; ERK42: 179.6 ± 7.5 %, *n* = 5 ) (Fig. 9b). In addition, COX2 has been reported to be implicated in long-term potentiation [40]. Here we demonstrated that there was no difference in the level of COX-2 normalized with GAPDH between vehicle control state and AUDA-treated groups (106.4 ± 13.2 % of vehicle, *n* = 3 ) (Fig. 9c) (*p* > 0.05).

## Discussion

In the present study, we provided evidence that sEHI enhanced the amplitude of evoked EPSCs and mEPSCs responses in PFC synapses by using whole-cell patch clamp recordings. Extracellular recordings consistency revealed increased fEPSPs and increased input-output plots in a dose dependent manner after AUDA treatment at PFC synapse from brain slices. Plasticity PPF is a neurotransmitter efficacy index of presynapse released probability [41]. An observed unchanged PPF, coupled with increased fEPSPs suggests alteration of postsynaptic glutamate neurotransmission in sEHI treated neurons. Moreover, sEHI facilitated a HFS-induced LTP. Finally, the protein levels of GluR1, GluR2, NR2A and NR2B were increased after treatment with sEHI AUDA. The AUDA further increases the LTP-induced ERK phosphorylation. These results provide new insights into the role that sEHI may play in the regulation of synaptic efficacy in the PFC area.

A report has demonstrated sEH specific expression in pyramidal neuron of layer V in the dorsomedial cerebral cortex [13]. It is still unknown how sEH exerts its effects in modulating the basal synaptic responses. Here we demonstrated that the sEH inhibitor, AUDA, induced the enhancement of synaptic neurotransmission. Enhanced levels of EETs resulting from sEH inhibition have been reported [42, 43]. We applied the 14, 15-EET also increases the fEPSPs response. The enhancement of postsynaptic response induced by sEH inhibitor, AUDA is blocked by selective EET antagonist. Thus sEH inhibitor causes the levels of EETs to increase and thereby enhances the synaptic excitatory response in the PFC region. Our results showed that the sEH inhibition enhanced the amplitude of evoked EPSCs and mEPSCs responses, but not PPF in PFC synapses. Moreover, sEH inhibition induced the increase of LTP magnitude in PFC synapses. These results suggest that sEH inhibition affects postsynaptic efficacy and is beneficial for maintaining LTP.

AMPA receptors and NMDA receptors have been considered as the major ionic glutamate receptors that are associated with excitatory neurotransmission and the mediated the LTP in the brain [22, 23, 44]. The synaptic response increment by sEH inhibition could be due to the increase of neurotransmitter release from presynaptic or increase in the number of glutamate postsynaptic receptors. Our electrophysiology data showed the PPF did not change by sEH inhibitor treatment, suggesting that sEH inhibitor did not alter the presynaptic neurotransmission at PFC synapse. Furthermore, we observed that inhibition of sEH at the PFC synapse enhances the protein levels of the NMDA receptor NR1 subunit, NR2A subunit and NR2B subunit; AMPA receptor GluR1 subunit, GluR2 subunit in the PFC region. Thus sEH inhibitor increases synaptic response mostly via altering the number of glutamate postsynaptic receptors. The altered protein levels of AMPA receptors and NMDA receptors by inhibiting of sEH did not enhance mRNA level of GluR1, GluR2, NR1 NR2A and NR2B in the PFC region. Our data further revealed that sEH inhibitor resulted in increased NR2B, GluR1 and GluR2 phosphorylation state, suggesting that post-translational modification is involved in sEH inhibitor altered synaptic response. The enhancement of phosphorylated protein levels is required for surface glutamate receptors stability. The phosphorylated protein of glutamate receptors also involved in regulation of NMDA receptors and AMPA receptors function [45, 46]. Further studies are needed to confirm how the phosphorylated protein in modulation of synaptic plasticity by sEH inhibition.

We investigated the molecular pathway under sEH inhibition treatment in PFC region. Previous studies demonstrated that sEH inhibition enhanced levels of EETs and increased PI3K/Akt and p42/p44 MAPK signaling in endothelial cells, cardiomyocytes and diabetic nephropathy disease [39, 42, 43, 47]. Additionally, MAPKs pathway and phosphorylated level of ERKs are classically required for NMDA-dependent LTP and long-term memory [48–52]. Our data showed that the phosphorylated level of ERKs was significantly increased after LTP. We also found that the sEH inhibitor AUDA not only induced an increase in phosphorylated ERKs in the mPFC slices, but also augmented LTP response, which further increased the phosphorylated ERK; thus, enhanced LTP and potentiated activation of ERKs which delivers glutamate receptors into the PFC post-synapse by treatment with sEH inhibition. Dopamine D2 receptors have been reported to be involved in cocaine facilitated protein synthesis dependent LTP [38, 48]. Our results demonstrated a sEHI AUDA-induced increase in dopamine D2 receptor activation in the PFC. Thus, the effects of sEHI AUDA could also be due to activation of D2 receptors that facilitates the formation and continuation of LTP. In addition, cyclooxygenase 2 (COX-2) has been reported to be required for synaptic plasticity and acquisition of memory process [40, 51]. We found that the protein level of COX-2, as detected by Western Blotting, showed no difference between sEH inhibition treatment and vehicle control in PFC slices. We know that the prefrontal cortex is important for emotional memory and experience memory [53–57]. Here we demonstrated that sEHI AUDA induced synaptic related molecular changes in NMDA receptor subunits NR1, NR2A and NR2B; and AMPA receptor subunits GluR1, GluR2; and in the dopamine D2 receptor. sEHI AUDA contributes to the degree of HFS-induced LTP in the PFC region. These findings could also suggest that synaptic properties of sEHI AUDA in PFC could lead to the modulation of learning and memory.

Additionally, animal studies have shown that acute or chronic pre-administration of sEH inhibitor could protect infarct size region and improve the neurodeficit score which damaged by cerebral ischemia [4, 58]. The glutamate transmission is dramatically increased during ischemia [59]. Preconditioning by treated with glutamate prevented oxygen-glucose deprivation (OGD)-induced neuronal injury blocked by AMPA receptor antagonist and NMDA receptor antagonist in primary cultured cortical neurons [60]. Our finding demonstrated that AUDA-induced the enhancement of glutamatergic neurotransmission in PFC area and other studies have shown OGD increased massive glutamatergic neurotransmission. Thus the underlying mechanisms of the neuronal protective effect of AUDA on stroke or how AUDA mediates regulation of vascular function or excitatory transmission still need to be assessed. The role of AUDA may regulate different responses underlie basal condition and pathological condition. In the present study, we observed that AUDA enhanced glutamatergic transmission and LTP in PFC area may be beneficial to learning and memory.

In summary, we showed that sEH inhibition induced an enhancement of PFC neuronal synaptic neurotransmission. This enhancement of synaptic neurotransmission is associated with an enhanced postsynaptic glutamatergic receptor and postsynaptic glutamatergic receptor mediated synaptic response. By contrast, the paired-pulse ratio is not changed after inhibition of sEH activity. The presynaptic mechanism is not involved in sEH inhibitor-induction of synaptic response. Furthermore, enhanced LTP may be via ERK phosphorylation resulting from the delivery of glutamate receptors into the PFC by post-synapse by treatment with AUDA. Our study provides a foundation for understanding PFC related synaptic efficacy and PFC related cognitive functioning with respect to learning and memory.

## Conclusions

We first report that sEH inhibitor mediated excitatory neurotransmission in the PFC area. Inhibition of sEH enhances LTP which through the ERK phosphorylation resulting from the delivery of glutamate receptors into the PFC area. It is suggested that inhibition of sEH may modulate the synaptic function and learning memory formation.
